# Review for the generalist: evaluation of anterior knee pain

**DOI:** 10.1186/1546-0096-5-8

**Published:** 2007-05-04

**Authors:** Kristin M Houghton

**Affiliations:** 1 Division of Rheumatology, British Columbia's Children's Hospital, K4-119 Ambulatory CareBuilding, 4480 Oak Street, Vancouver British Columbia, Canada V6H 3V4 and Allan McGavin Sports Medicine Centre, University of British Columbia, 3055 Wesbrook Mall, Vancouver British Columbia, V6T 1Z3, Canada

## Abstract

Anterior knee pain is common in children and adolescents. Evaluation and management is challenging and requires a thorough history and physical exam, and understanding of the pediatric skeleton. This article will review common causes of chronic anterior knee pain in the pediatric population with a focus on patellofemoral pain.

## Background

**Figure 1:**
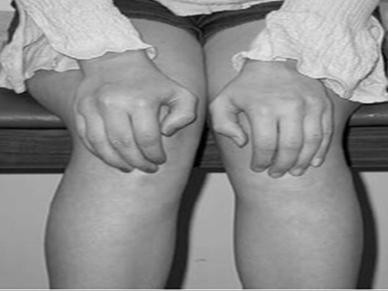
Patients with anterior knee pain localize the pain to the anterior knee and will point to the entire extensor mechanism, rather than to specific anatomic regions [1].

Anterior knee pain (see Figure 1) is one of the most common musculoskeletal complaints seen in the pediatric population. A fairly extensive differential diagnosis exists as anterior knee pain is a fairly nonspecific phenomenon. A thorough history and physical examination with attention to anatomic location of the pain, inciting factors, relationship of associated symptoms as well as a general assessment of growth and development will aid in the evaluation and treatment of this disorder. An awareness of typical injury patterns can aid the physician in narrowing the differential diagnosis. Identification of worrisome signs and symptoms will also help in defining which cases may require further evaluation. All pediatric patients presenting with knee pain require evaluation for ipsilateral hip and lumbar spine disorders. This article will review common causes of chronic anterior knee pain in the pediatric population with a focus on patellofemoral pain. It is not meant to be an exhaustive review and will not review acute traumatic knee injuries. Lateral, medial or posterior knee pain will be covered in a subsequent article.

### Clinical history

The clinical history should include a thorough description of the pain characteristics (location, character, onset, duration, change with activity or rest, aggravating and alleviating factors, night pain); trauma (acute macrotrauma, repetitive microtrauma, recent/remote); mechanical symptoms (locking or extension block, instability, worse during or after activity); inflammatory symptoms (morning stiffness, swelling); effects of previous treatments and the current level of function of the child. Patients with overuse anterior knee pain may report a sensation of giving way or instability. This sensation is usually a pseudo-giving way due to what is coined the "quadriceps inhibitory reflex" or a neuromuscular inhibition that occurs secondary to pain, muscle weakness, patellar instability or joint instability. It is important to note that true instability is suggestive of internal derangement of the knee most commonly associated with anterior cruciate ligament (ACL) tears or meniscal injury [1].

A history of previous injury or knee surgery, chronic inflammatory joint disease or bleeding diathesis, is significant, especially if knee swelling is present.

### Physical examination

**Figure 2:**
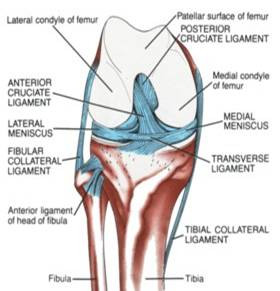
**A) **Anterior anatomy of the knee. (Picture courtesy of Allan McGavin Sports Medicine Centre, Vancouver, Canada).

The basic anatomy of the knee is shown in Figure 2. The knee is the largest joint in the body and is comprised of the patellofemoral joint, medial tibiofemoral joint, lateral tibiofemoral joint and superior tibiofibular joint. It is a modified hinge joint with primary motion in the sagittal plane (flexion – extension). During the clinical assessment the physician should try and reproduce the patient's knee pain through palpation as well as biomechanical evaluation. Biomechanical examination is important in determining any potential predisposing or contributing factors. This should include an assessment for genetic predisposing factors such as excessive stiffness, loose-jointedness, and/or poor muscle tone. Functional biomechanics should be assessed by evaluation of gait, and maneuvers such as jumping, hopping (single and double-leg) and squatting. Knee swelling is unusual in anterior knee pain and generally implies intra-articular pathology, synovitis or loose body. The contralateral knee and ipsilateral hip should always be examined [2,3].

### Physical examination in patients with anterior knee pain

#### 1. Observation

**Figure 3:**
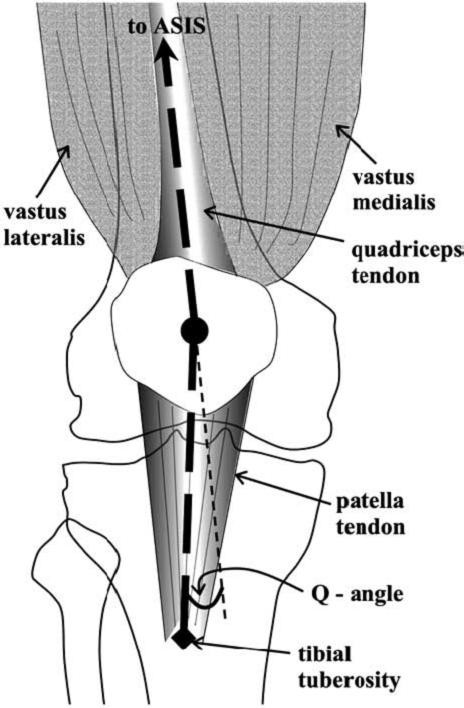
The quadriceps "Q" angle is formed between the line joining the anterior superior iliac spine (ASIS) and the centre of the patella and the line joining the centre of the patella and the tibial tuberosity [6].

Standing [pelvis heights, lower limb alignment, "Q" quadriceps angle (formed by the intersection of the line drawn from the anterior superior iliac spine to the centre of the patella and the line drawn from the centre of the patella to the tibial tuberosity. Normal Q angles are 8 to 12° for males and 15 to 18° for females) [4,5], patellar alignment, muscle asymmetry or wasting]. (See Figure 3) [6].

Supine [lower limb lengths and alignment, patellar position, patellar tilting or rotation]

Squat [patellar tracking and "J" sign: medial shift of the patella as it enters the trochlea in early flexion, suggestive of vastus medialis obliqus (VMO) weakness or tight lateral retinaculum]

Gait [lower limb alignment, hip abduction weakness, subtalar pronation]

#### 2. Range of motion

Active movements [knee flexion (135°), knee extension (0°), internal and external rotation (10°), isometric quadriceps contraction]

Passive movements [patellar tilt, lateral tracking of patella or increased Q angle]

#### 3. Palpation

Surface anatomy is best appreciated with the knee flexed to 90 degrees. The point of maximal tenderness should be correlated with the underlying bone or soft tissue anatomy. Palpate and note tenderness over the patellofemoral joint, bones, menisci and along the course and attachments of ligaments and tendons. Patients with patellofemoral pain typically have tenderness over the medical and/or lateral patellar facets, superior and inferior poles of the patella or patellar tendon. Pain over the physes or joint line is not part of patellofemoral pain.

Effusion [may be visible or palpable. In situations where there is minimal fluid, compression of the suprapatellar pouch may decrease the joint volume and aid detecting an effusion].

#### 4. Special tests

1) Patellar tilt test [patient supine and knee extended – apply downward pressure to the medial edge of the patella to assess lateral patellar restraints. Test is positive if the patellar tilt angle is more than 5 degrees.]

2) Medial and lateral patellar glide [push patella medially and laterally noting degree of movement relative to width of patella, > 75% translation is hypermobile]

3) Quadriceps setting/grind test, patellar inhibition [suprapatellar resistance while patient performs isometric quadriceps contraction with knee in full extension. Pain indicates a positive test.]

4) Patellar compression test [direct compression of the patella into the trochlea, pain indicative of articular cartilage degeneration or acute chondral injury]

5) Patellar apprehension test [apply lateral pressure to patella at 30° knee flexion, positive if patient feels instability or pain]

6) Lachman test [positive for anterior cruciate insufficiency if there is anterior translation of tibia on femur with knee in 30° flexion]

7) Posterior drawer test [positive for posterior cruciate ligament insufficiency if there is posterior translation of tibia on femur with knee in 90° flexion]

8) McMurray's test [positive for meniscal tear if palpable or audible click. Patient supine, flex hip to 90°, maximally flex knee followed by passive knee extension, first with external tibial rotation while applying a varus stress at the knee (lateral meniscus) and then with internal tibial rotation while applying a valgus stress (medial meniscus) at the knee]

9) Trendelenberg test [positive when patient stands on one leg and contralateral hip drops, indicative of gluteal/hip abductor weakness]

#### 5. Flexibility

The knee should be moved thru active range of flexion and extension and then placed thru full passive range of motion. Active range of knee motion includes flexion (135°), extension (0°), internal and external rotation (10°). Muscles that span two joints are important for functional range of motion and should be tested independently (hamstrings, rectus femoris, gracilis, tensor fascia lata and iliotibial band, and gastrocnemius muscles).

#### 6. Strength

Knee extension and flexion

Hip extension, flexion, abduction, adduction.

#### 7. Hip joint and lumbar spine exam

Range of motion. Further detailed exam as appropriate.

#### 8. Neurovascular exam

Patellar reflex [L2, L3, L4]

Sensation of lower leg in major dermatomes

Popliteal and dorsalis pedis artery pulsations

### Investigations

Laboratory tests are almost never necessary in evaluating a patient with anterior knee pain. CBC, ESR or CRP, blood and joint cultures should be done if infection is suspected. Arthritis is a clinical diagnosis; ANA, rheumatoid factor and HLA-B27 are helpful in classification and treatment but not diagnosis.

### Imaging

Radiographs and magnetic resonance imaging (MRI) are the most common imaging tools used to assess the pediatric knee. As with all tests, false positive and false negative results may occur and clinical correlation is necessary.

#### Radiographs

Routine radiographs of the knee include weight-bearing anteroposterior (AP) and lateral views. The AP view allows evaluation of the distal femoral physis, proximal tibial physis, tibial intercondylar eminence, and patella. The lateral view allows evaluation of the position of the patella and tibial tubercle. Axial (skyline patellar profile) and tunnel (notch) views are part of the traditional orthopedic evaluation. The axial view is tangential to the anterior aspect of the flexed knee (usually 30 to 45 degrees of knee flexion) allowing evaluation of the congruity of the patellofemoral articulation and a measure of patellar tilt. The tunnel (notch) view, most commonly used in pediatrics to diagnose osteochondritis dissecans, is an AP radiograph with the knee flexed to 20 degrees to allow evaluation of the articular surfaces of the distal femoral condyles [7].

#### Magnetic Resonance Imaging (MRI)

MRI is a powerful tool in diagnosing knee disorders. In the absence of established guidelines, it is commonly misused as a screening examination. MRI images are obtained in axial, coronal and sagittal planes. MRI allows evaluation of the soft tissues including the muscles, tendons, menisci, cruciate and collateral ligaments, retinaculum, neurovascular bundle, bursa and synovium. MRI also allows a more detailed evaluation of osseous structures including articular and physeal cartilage, subchondral bone, periosteum, and bone marrow elements. Intravenous injection of gadolinium is not routine but is useful to delineate synovial enhancement [8].

MRI should be done only after a thorough clinical examination and standard radiographs are unable to provide a specific diagnosis. Ideally it should be ordered by a physician capable of interpreting the images and providing definitive treatment.

### Common causes of chronic anterior knee pain

Pain receptors are present within several knee structures, including the patella, synovium, fat pad, tendon, subchondral bone, and quadriceps retinaculum [9-11]]. Any of these structures, individually or in combination, can cause anterior knee pain. Most causes of anterior knee pain involve the patellofemoral joint and the extensor mechanism of the knee. The term anterior knee pain is often used interchangeably with patellofemoral pain. Previous literature has also interchanged the term "chondromalacia patella" with anterior knee pain but this is now out of favor; chondromalacia patella is a surgical finding not a diagnosis. Patients with anterior knee pain do not necessarily have chondromalacia patella and patients with chondromalacia do not uniformly have pain. Anterior knee pain may be caused by conditions unique to the growing pediatric skeleton including hip disease (slipped capital femoral epiphyses and Perthe's disease) or osteochondroses (Osgood-Schlatter disease or Sinding-Larsen-Johansen disease). Anterior knee pain is occasionally caused by serious underlying systemic disease including inflammatory conditions and malignancies.

### Patellofemoral Pain Syndrome

Patellofemoral pain (PFP) is the most common knee problem presenting to primary care physicians. Patellofemoral syndrome is a non-specific descriptive term used to describe pain in and around the patella.

#### Epidemiology

PFP affects active and non-active children, is more common in girls and is most common during the adolescent growth spurt. Up to 30% of all visits to sports medicine practitioners are for anterior knee pain. Incidence rates of 7% and 10% have been reported in young male and female athletes respectively [12]. Symptoms usually affect both knees with one side more symptomatic than the other.

#### Etiology

The etiology of PFP is unclear. There are currently two main theories: malalignment of the patella relative to the femoral trochlea with resultant articular cartilage abnormalities, and excessive mechanical loading and chemical irritation of local nerve endings with resultant peripatellar synovitis [13,14]. Soft tissue tightness is common during the adolescent growth spurt with resultant inflexibilities altering the stress through the patellofemoral joint. Acute trauma, repetitive microtrauma/overuse and malalignment all can lead to increased strain on the peripatellar soft tissues or increased patellofemoral joint stress.

#### Anatomy and biomechanics

The patella functions to facilitate knee extension and increases the force of extension by up to 50% [15]. It centralizes the forces of the quadriceps, helps transmit the force to the patellar tendon and tibial tuberosity, and also protects the patellar tendon from contact with the femur. The patellofemoral joint reaction force (PFJR) acts perpendicular to the articular surface and increases with increasing knee flexion. PFJR is calculated to be 0.5 times body weight while walking, 3 to 4 times when climbing stairs, 7 to 8 times when squatting and 20 times body weight when jumping [16]. The patella changes its orientation during movement. Patellar tracking is determined by both static (medial and lateral retinaculum, bony architecture of the trochlea) and dynamic forces (quadriceps) acting on the patella, the congruity of the patellofemoral articular surfaces and the alignment and biomechanics of the lower extremity.

#### Risk factors

Increased patellofemoral joint stress and altered patellar tracking may increase the risk for PFP. Direct trauma to a flexed knee may disrupt the articular cartilage and repetitive microtrauma from chronic overloading (weight bearing exercise) may increase patellofemoral stress.

Malalignment of the lower extremity influences patellar tracking and may include genu valgum, genu varum, genu recurvatum, leg length discrepancy, femoral anteversion, external tibial torsion, lateral displacement of the tibial tubercle, and excessive pronation of the subtalar joint [14]. Larger Q angles (genu valgum, femoral anteversion, external tibial torsion and subtalar joint pronation) are associated with increased static patellofemoral joint stress but clinical studies fail to demonstrate a consistent correlation between larger Q angles and PFP [14]. Lateral displacement of the tibial tubercle greater than 10° from the midpoint of the patella may cause lateral tracking of the patella and PFP [14].

Quadriceps muscle weakness or imbalance with relative vastus medialis (VMO) weakness or imbalance in neuromuscular control of the VMO and vastus lateralis (VL) may cause PFP. The VMO is the weakest portion of the quadriceps, the first to atrophy with disuse and the last to rehabilitate. VMO weakness results in lateral shifting and tracking of the patella during the last 30° of extension with resultant reduction in patellofemoral contact area and increased patellofemoral stress. Weakness of the gluteal muscles can also influence patellar tracking as they assist in extension of the knee by virtue of their insertion into the iliotibial tract, in addition to their primary function to extend, abduct and externally rotate the hip.

Muscle inflexibilities can contribute to PFP. Tight quadriceps are less able to absorb eccentric loads and offload stress to the quadriceps, patellar tendons and hamstrings, thus increasing the PFJR. Inflexibility of the gastrocnemius leads to increased subtalar pronation which may influence patellar tracking. Hamstring tightness also increases the PFJR; an associated knee flexion contracture is common in adolescents during rapid growth. Hip flexion contractures cause a functional knee flexion contracture which also increases the PFJR. Tightness of the lateral retinaculum, iliotibial band and lateral structures can cause increased lateral tracking of the patella and excessive stress on the lateral patellofemoral joint.

Patients with PFP may have decreased proprioception although it is unclear whether decreased proprioception is causative of or a result of patellar maltracking [17,18].

Congenital anomalies of the patella or trochlea associated with PFP include hypoplasia of the medial patellar facet, hypoplasia of the medial trochlea and flattening of the trochlear groove, hypoplasia of the lateral femoral condyle, patella alta, and patella inferna [14].

#### Clinical history

The most common presenting complaint is dull and achy knee pain during and after activity, pain after prolonged sitting with the knee in flexion ("theatre sign") and stiffness. Pain may be peripatellar or retropatellar. Aggravating activities include weight bearing sport, stairs (descending worse than ascending) and squatting. Swelling is unusual. True instability does not occur but patients often report "giving way" due to reflex inhibition of the quadriceps muscle secondary to pain, effusion or deconditioning. Historical clues to differentiate instability from "giving way" include the occurrence of instability (meniscal or ligament injury) with pivoting or twisting versus the occurrence of "giving way" with increased PFJR such as ascending or descending stairs or walking on an incline.

#### Physical exam

Lower extremity alignment should be noted. There may be pain with squatting. VMO wasting may be marked. There may be variable superolateral or inferomedial retinacular or patellar facet tenderness. The lateral soft tissue patellar restraints are often tight with decreased passive medial patellar glide. The quadriceps setting/grind test is the most specific test with 96% specificity and 40% sensitivity [19].

#### Management

Reassurance and education are the most important components of multimodal therapy. Gradual improvement and resolution of symptoms is the rule. Other therapies include: activity modification (decrease forces across patellofemoral joint), flexibility exercises (hamstrings, quadriceps, iliotibial band, lateral retinaculum, gastrocnemius), strengthening of the vastus medialis, patellar tracking exercises, cryotherapy, short term NSAID therapy, patellofemoral orthoses or patellar taping, and shoe orthoses (subtalar pronation). Any training errors should also be addressed. General return to play guidelines can be followed for PFP and most causes of anterior knee pain (see Table 1).

**Table 1:** Return to play guidelines

1. Control pain and inflammation	PRICE (protect, rest, ice, compression, elevation)
2. Optimize range of motion	Active and passive exercises
3. Optimize strength	Isometric, isotonic exercises
4. Optimize proprioception	Balance, patellar tracking exercises
5. Functional and sport specific skills	Running, jumping, pivoting etc.
6. Return to usual activities or sport	Practice before competitive play

### Patellofemoral instability and patellar subluxation

Patellofemoral dysplasia, whether congenital or acquired, represents a spectrum of disorders characterized by static and dynamic malalignment of the patella relative to the distal femur. Malalignment results in maltracking of the patella with resultant instability and potential osteochondral injury. Four clinical subtypes are recognized based on the degree of malalignment: mild dynamic imbalance of the patella with excessive loading of the lateral patellar and femoral facets (lateral patellar compression syndrome); chronic subluxing patella; recurrent patellar dislocation; and chronic (or obligatory) dislocation of the patella. Patients with patellofemoral instability may complain of anterior knee pain, episodic giving way, locking and catching sensations associated with recurrent effusions. On examination, findings are similar to PFP. Patella alta, tenderness over the medial patella femoral ligament, positive patellar apprehension test or frank lateral dislocation may be elicited. Treatment includes stretching tight lateral structures (vastus lateralis, iliotibial band), strengthening medial structures (VMO), knee orthoses for patellar stabilization, and foot orthoses to correct excessive subtalar pronation. Orthopedic referral and possible surgical management is recommended for recurrent and chronic obligatory patellar dislocation.

### Patellar tendinopathy

"Jumper's knee" or patellar tendinopathy is a common cause of infrapatellar pain. Pain is usually maximal at the patellar attachment and proximal tendon and is aggravated by jumping or hopping. In children and adolescents the osteochondroses/traction apophysitis Osgood-Schlatter (OSD) and Sinding-Larsen-Johansson (SLJD) disease commonly present with pain at the attachments of the patellar tendon. (See osteochondroses section below) PFP is commonly associated with patellar tendinopathy. On examination there is local tenderness over the patellar tendon; thickening or nodules may be palpable. There may be abnormalities of the PF joint. Treatment requires load reduction (activity modification, biomechanical correction), cryotherapy and progressive eccentric strengthening.

### Osteochondroses

**Figure 4:**
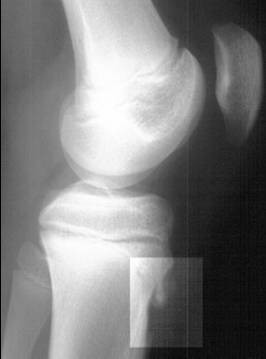
Osgood-Schlatter disease. Lateral radiograph of the knee demonstrating fragmentation of the tibial tubercle with overlying soft tissue swelling. (Radiograph courtesy of BC Children's Hospital)

Osgood-Schlatter (OSD) and Sinding-Larsen-Johansson (SLJD) disease are osteochondroses and traction apophysitis affecting the extensor mechanism of the knee. OSD occurs at the growth plate of the tibial tuberosity at the inferior attachment of the patellar ligament and is more common than SLJD, which occurs at the inferior pole of the patella at the superior attachment of the patellar ligament. Both conditions present in adolescence during the growth spurt and are characterized by well localized pain aggravated by exercise. On examination there is well localized tenderness and soft tissue swelling. There is often tightness of surrounding muscles, especially the quadriceps, hamstrings and gastrocnemius. Both OSD and SLJD are clinical diagnoses and radiographs are not usually required. If pain is severe or there is marked swelling radiographs should be obtained to exclude bony tumors or infection. Typical radiographs of OSD show enlargement and fragmentation of the tubercle (Figure 4). Management is conservative; these are self-limited conditions that resolve with skeletal maturity but symptoms may persist for up to 24 months. Treatment includes activity modification, local cryotherapy, local muscle stretching and strengthening, and correction of any predisposing biomechanical factors, such as subtalar pronation with physical therapy, bracing and/or orthotics. Occasionally in OSD, symptoms may persist with skeletal maturity due to non-union of the tibial tuberosity. The separate fragment should be excised.

### Fat pad irritation/impingement

The infrapatellar fat pad is richly innervated and injury may cause anterior knee pain. Impingement of the infrapatellar fat pad between the patella and the femoral condyle may be secondary to direct trauma or acute hyperextension injury. Chronic irritation may be associated with patellar tendinopathy, patellofemoral syndrome or exposure to recurrent synovitis. Pain is often worsened by knee extension, prolonged standing and kneeling. On examination there is localized tenderness and swelling in the fat pad with posterior displacement of the inferior pole of the patella. Associated predisposing biomechanical factors include genu recurvatum and anterior tilting of the pelvis. Treatment includes local cryotherapy, taping the patella to reduce the amount of tilt and impingement, local muscle flexibility and strengthening, and correcting lower limb biomechanics as in patellofemoral syndrome.

### Referred pain from the hip or lumbar spine

All patients with knee pain require evaluation of their hips and lumbar spine. Children with Perthe's disease or slipped capital femoral epiphysis (SCFE) may present with knee pain. Perthe's disease, an idiopathic avascular necrosis/osteonecrosis of the femoral epiphysis usually affects 4 to 10 year olds, is more common in boys and is bilateral in 10%. Children usually present with a limp or pain in the hip, thigh or knee. Examination of the knee is normal but there is limited internal rotation and abduction of the ipsilateral hip. Trendelenberg may be positive. Radiographs vary with the stage of the disease but may show evidence of bone necrosis, fragmentation, reossification or remodeling and healing. Treatment consists of rest from aggravating activities and range of motion exercises. Occasionally orthoses or surgery may be required. Generally, the condition resolves and children return to their activities. The main long-term concern is early osteoarthritis.

SCFE, displacement of the proximal femoral epiphysis on the femoral neck, usually affects 10 to 15 year olds and is more common in boys. Adolescents usually present with a limp and may have hip, groin or knee pain. The knee exam is normal, the hip is often preferentially held in abduction and external rotation with decreased active and passive internal rotation and adduction. Trendelenberg may be positive. Radiographs (anterior-posterior and frog leg lateral) of the hip may show widening and irregularity of the physis with posterior inferior displacement of femoral head. SCFE may compromise the vascular supply to the femoral head and lead to avascular necrosis; all cases warrant orthopedic referral. Treatment includes non weight bearing, traction and surgery with epiphyseal fixation and osteotomy. Most patients do well after surgical fixation. Complications include avascular necrosis and chondrolysis. Patients require long term follow up as SCFE may develop within 12 to 18 months in the contralateral hip, if prophylactic pinning is not performed [20].

### Osteochondritis Dissecans

Osteochondritis dissecans (OCD) may affect the knee; most commonly the lateral aspect of the medial femoral condyle but also the lateral femoral condyle or patella. OCD is an idiopathic lesion of bone and cartilage resulting in bone necrosis and loss of continuity with subchondral bone. There may be partial or complete separation of articular cartilage with or without involvement of subchondral bone. It occurs in adolescence and lesions are bilateral in 20%. Proposed etiologies include acute trauma, repetitive microtrauma, vascular insufficiency or normal growth variant [1]. Adolescents may present with activity related pain and swelling. Locking and catching due to fragment instability is uncommon.

**Figure 5:**
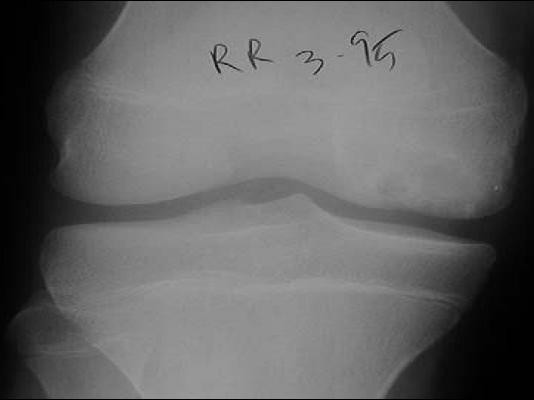
Osteochondritis dissecans. Anterior posterior  radiograph shows minimal cystic changes affecting the lateral aspect of  the medial femoral condyle [21].

**Figure 6:**
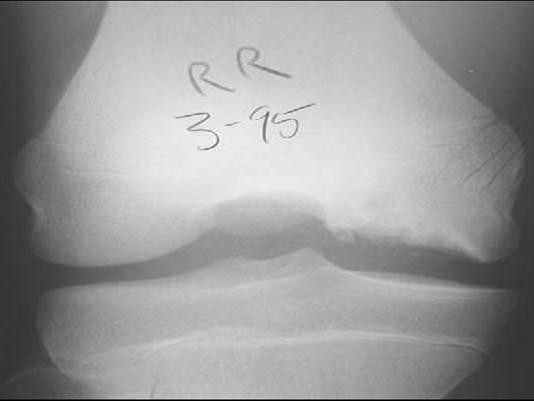
Osteochondritis dissecans. Tunnel or notch views show the cystic changes more clearly [21].

On examination, there may be focal bony tenderness, knee effusion and evidence of a loose fragment with extension block or palpable loose body. Wilson's sign is seen in medial femoral condyle lesions. (Wilson's sign is positive if pain is reproduced by flexing the knee to 90° and rotating the tibia medially while extending the knee. Pain occurs at approximately 30° and is relieved by lateral rotation of the patella.) Radiographs may show a radiolucent lesion, subchondral fracture, potential separation with subchondral bone and a loose body. OCD lesions can be missed on routine non weight bearing anterior/posterior and lateral radiographs; tunnel (notch) and axial (skyline patellar profile) views are required to view the articular surfaces of the distal femoral condyles and patella (See Figures 5 and 6) [21]. MRI may show cartilage changes earlier with contrast enhancement of intact cartilage lesions [21].

Treatment depends on the stage of the lesion and the skeletal maturity of the patient with early lesions in skeletally immature patients having the best prognosis. Skeletally mature patients or patients presenting with a locked knee or symptomatic loose body require urgent orthopedic evaluation. Treatment options include modified activity with or without weight-bearing; immobilization; cryotherapy; anti-inflammatories; drilling of subchondral bone to improve vascularity; reattachment and or removal of loose bodies and OATS procedure (osteochondral allograft transplant) [22].

### Synovial plica

The knee has normal synovial folds that are residual embryonic remnants persisting from when the knee cavity was a septated structure. Occasionally, pathological conditions occur within the plica(e) due to local synovitis caused by acute, direct or repetitive microtrauma. The inflamed plica can become trapped between the patella and the femoral condyle. Impingement can cause further inflammation and fibrosis with abrasion of the femoral condyle, changes in the articular surface and reactive changes in the subchondral bone. Diagnosis is clinical; common complaints are of medial knee pain worsened with running, stair climbing or squatting accompanied by snapping and catching during flexion (usually flexion arc 30–70 degrees). On examination the medial patellar retinaculum from the patella to the medial femoral condyle or anteromedial joint line may be tender. The plica may be palpable as a thickened band that is tender when pressed against the edge of the condyle. Management includes patellar mobilization and massage, flexibility exercises for the hamstrings, quadriceps and gastrocnemius, and anti-inflammatory medications with surgical removal of the plica reserved for recalcitrant symptoms.

### Quadriceps tendinopathy

Pain arising at the quadriceps tendon attachment to the patella is uncommon in the pediatric population. On examination there is tenderness at the superior margin of the patella and pain is exacerbated by resisted quadriceps contraction. Treatment includes local massage at the site of pain in addition to the general principles used for patellar tendinopathy.

### Bipartite patella

The patella ossifies between 3 and 5 years of age with gradual coalescence of multiple ossification centers. Accessory ossification centres often occur; the most common location is the superolateral patella. A bipartite patella is usually an asymptomatic, incidental radiographic finding. Radiographs show regular smooth fragment margins with no evidence of soft tissue swelling, differentiating the lesion from acute fracture. Occasionally bipartite patella may cause anterior knee pain. Clinically there is soft tissue tenderness at the superolateral pole of the patella. Initial treatment is conservative with modified activity and local flexibility and strengthening exercises. Surgical options include excision or fixation of the bipartite patella or release of the vastus lateralis insertion [2].

### Stress fracture of the patella

Stress fractures of the patella are rare causes of anterior knee pain. In the pediatric population, it is reported in jumping athletes who present with intense localized pain with possible overlying soft tissue swelling. Radiographs will detect chronic stress reaction; early lesions are best detected by radionucleotide bone scan.

### Bursitis

There are multiple bursas around the knee. Bursa are synovial lined structures that provide a low-friction interface between tissues to provide smooth motion. Bursitis, inflammation of the bursa, can be secondary to repetitive microtrauma from overuse, direct trauma, autoimmune disease including juvenile idiopathic arthritis (JIA) and systemic lupus erythematosus (SLE), or infection. Prepatellar bursitis is the most common bursitis of the knee, presenting with anterior knee pain and swelling. Infrapatellar bursitis may also cause anterior knee pain mimicking patellar tendinopathy but can be diagnosed by careful palpation for localized tenderness. In both conditions it is important to differentiate fluid in the bursa from intraarticular effusion. Treatment includes local cryotherapy, nonsteroidal anti-inflammatory medications (NSAID's) and avoidance of further direct trauma to the bursa. If there are any signs of septic bursitis the bursa should be aspirated and appropriate antibiotics prescribed.

### Inflammatory disorders

Oligoarticular JIA often presents with monoarticular knee arthritis. Children may present with anterior knee pain but the typical history of morning stiffness, gradual resolution of pain with activity and clinical exam findings usually allow the practitioner to make the correct diagnosis. A complete joint and systemic examination to exclude other joint involvement is important as is screening for asymptomatic uveitis associated with JIA.

The JIA subtype, Enthesitis Related Arthritis, is unique in that it is more common in school-age boys, is frequently associated with the genetic marker HLA-B27 and inflammation of the entheses may be a prominent finding. Enthesitis (inflammation at the attachment of tendons, ligaments and fascia to bone with characteristic pain on palpation) around the knee may be confused with Osgood's Schlatter or Sinding-Larsen Johannsen disease. Enthesitis is usually associated with arthritis and symptoms are more prominent in the morning, which helps to differentiate it from the osteochondroses and traction apophysitis group.

Treatment of arthritis includes physiotherapy for improving range of motion and strength, NSAID's and potentially local intra-articular corticosteroid injection or disease modifying anti-rheumatic therapy. All children and adolescents suspected of having JIA or other chronic inflammatory arthritis should be referred to a pediatric rheumatologist.

### Pain amplification syndromes

Many pediatric patients with severe chronic musculoskeletal pain do not have an identified cause. The cause of amplified musculoskeletal pain is unknown, but minor trauma, underlying chronic illnesses and psychological distress have been associated. This condition has been called many terms including reflex sympathetic dystrophy (RSD), reflex neurovascular dystrophy (RND), complex regional pain syndrome, and chronic musculoskeletal pain syndrome with or without autonomic dysfunction. Typically, an affected child sustains minor trauma to the lower extremity which results in pain out of proportion to the injury.

On physical examination, patients may be unwilling to weight bear, manifest allodynia (pain generated by normally non-painful stimuli), and autonomic changes such as excessive perspiration, edema, cyanosis, mottling, and coolness of the skin. General and neurovascular exam are normal.

Diagnostic studies are typically not helpful. X-rays may show osteopenia but only in chronic cases with significant disability. MRI can show regional bone marrow edema, and a technetium bone scan may show decreased uptake [23].

### Tumors

Benign and malignant tumors are rare causes of knee pain. Symptomatic benign bone lesions include osteochondroma, nonossifying fibroma with stress fracture, osteoid osteoma and chondroblastoma. Malignant bone tumors, include local osteosarcoma or Ewing's sarcoma, leukemia or metastases from neuroblastoma. Benign synovial tumors include pigmented villonodular synovitis (PVNS) and hemangioma. Malignant synovial tumors include sarcomas.

## Conclusion

Anterior knee pain is common in children and adolescents. Evaluation and management is challenging and requires a thorough history and physical exam, and understanding of the pediatric skeleton. Most causes of anterior knee pain involve the patellofemoral joint and the extensor mechanism of the knee. It is important for the clinician to consider conditions unique to the growing pediatric skeleton including hip disease (slipped capital femoral epiphyses and Perthe's disease) or osteochondroses (Osgood-Schlatter disease or Sinding-Larsen-Johansen disease). Anterior knee pain is occasionally caused by serious underlying systemic disease including inflammatory conditions and malignancies. The clinical history and physical exam often lead to correct diagnosis and identify cases requiring further evaluation, imaging or referral. General return to play guidelines can be followed for PFP and most causes of anterior knee pain

## Abbreviations

ACL = anterior collateral ligament

AP = anteroposterior

JIA = juvenile idiopathic arthritis

MRI = magnetic resonance imaging

NSAID('s) = non-steroidal antinflammatory drugs

OATS = osteochondral allograft transplant

OCD = osteochondritis dissecans disease

OSD = Osgood-Schlatter's disease

PFJR = Patellofemoral joint reaction force

PFP = Patellofemoral pain

PVNS = pigmented villonodular synovitis

RND = Reflex neurovascular dystrophy

RSD = Reflex sympathetic dystrophy

SCFE = Slipped capital femoral epiphysis

SLE = systemic lupus erythematosus

SLJD = Sinding-Larsen-Johansson

VL = vastus lateralis

VMO = vastus medialis obliqus
